# Prevalence of strabismus and its risk factors among school aged children: The Hong Kong Children Eye Study

**DOI:** 10.1038/s41598-021-93131-w

**Published:** 2021-07-05

**Authors:** Xiu Juan Zhang, Yi Han Lau, Yu Meng Wang, Ka Wai Kam, Patrick Ip, Wilson W. Yip, Simon T. Ko, Alvin L. Young, Clement C. Tham, Chi Pui Pang, Li Jia Chen, Jason C. Yam

**Affiliations:** 1 Department of Ophthalmology and Visual Sciences, The Chinese University of Hong Kong, 4/F, Hong Kong Eye Hospital, 147K Argyle Street, Kowloon, Hong Kong SAR China; 2 Department of Ophthalmology and Visual Sciences, Prince of Wales Hospital, Sha Tin, Hong Kong SAR China; 3 Department of Paediatrics and Adolescent Medicine, LKS Faculty of Medicine, The University of Hong Kong, Pok Fu Lam, Hong Kong SAR China; 4 Department of Ophthalmology, Tung Wah Eastern Hospital, So Kon Po, Hong Kong SAR China; 5 Hong Kong Eye Hospital, Kowloon, Hong Kong SAR China; 6 Department of Ophthalmology, Hong Kong Children’s Hospital, Ngau Tau Kok, Hong Kong SAR China

**Keywords:** Eye diseases, Risk factors

## Abstract

The study aims to determine the prevalence of strabismus and its risk factors among school children in Hong Kong. This is a cross-sectional study involving 6–8 year old children from different districts in Hong Kong. 4273 children received comprehensive ophthalmological examination, cycloplegic auto-refraction, best corrected visual acuity (BCVA), anterior segment examination, cover/uncover test, ocular motility, and fundus examination. Demographic information, pre- and post- natal background, parental smoking status, and family history of strabismus were obtained through questionnaires. Strabismus was found among 133 children (3.11%, 95% CI 2.59–3.63%), including 117 (2.74%) exotropia and 12 (0.28%) esotropia cases (exotropia-esotropia ratio: 9.75:1). There was no significant difference in prevalence across age (6–8 years) and gender. Multivariate analysis revealed associations of strabismus with myopia (≤ − 1.00D; OR 1.61; 95% CI 1.03–2.52; *P* = 0.037) hyperopia (≥ + 2.00D; OR 2.49; 95% CI 1.42–4.39; *P* = 0.002), astigmatism (≥ + 2.00D; OR 2.32; 95% CI 1.36–3.94; *P* = 0.002), and anisometropia (≥ 2.00D; OR 3.21; 95% CI 1.36–7.55; *P* = 0.008). Other risk factors for strabismus included maternal smoking during pregnancy (OR 4.21; 95% CI 1.80–9.81; *P* = 0.001), family history of strabismus (OR 6.36; 95% CI 2.78–14.50, *P* < 0.0001) and advanced maternal age at childbirth (> 35 years; OR 1.65; CI 1.09–2.49, *P* = 0.018). The prevalence of strabismus among children aged 6—8 years in Hong Kong is 3.11%. Refractive errors, family history of strabismus and maternal smoking history during pregnancy are risk factors. Early correction of refractive errors and avoidance of maternal smoking during pregnancy are potentially helpful in preventing strabismus.

## Introduction

Strabismus is an important cause of amblyopia and other visual impairments among school children^1^. It is associated with reduced visual function and eye-related quality of life in children and also affects quality of life of their parents^2^. Early detection and appropriate intervention can improve binocularity and prevent occurrence of permanent visual deficits^3^.

Epidemiologic studies on strabismus and its risk factors among preschool and school-aged children have been conducted in different populations of different living environments. The Multi-Ethnic Pediatric Eye Disease Study (MEPEDS) included children of Asian and Caucasian descent in the United States^1,4^. Similar strabismus prevalence was reported among Hispanic/Latino (2.4%) and African-American children (2.5%)^1^. The Baltimore Pediatric Eye Disease Study (BPEDS) was on white and African American children^3^. The prevalence of strabismus was found to be 3.3% and 2.1% among White and African Americans respectively. The Strabismus, Amblyopia, and Refractive Error in Singaporean Children Study (STARS) was conducted in Singapore among children of dominant Chinese ethnicity^5^. According to their study, the prevalence of strabismus among children (6 to 72 months) was found to be 0.84%. Notably, their exotropia to esotropia (7:1) was similar to that our data (9.75:1). Similar study conducted in Asia, the Korea National Health and Nutrition Examination Survey detected a strabismic prevalence of 1.6% among children and adolescents aged from 5 to 18^6^. In Hong Kong, epidemiological data on strabismus is not known although it is needed for health care policies. It is expected to be different from other populations due to differences in ethnicity and environmental factors^1,4^. In this study, we aim to determine the prevalence and risk factors of strabismus in Chinese children aged 6–8 years in Hong Kong.

## Methods

### Study design and population

The study subjects were recruited from the Hong Kong Children Eye Study (HKCES)^7–10^, which is a population-based cohort study of eye conditions among children aged 6–8 years old in Hong Kong. The HKCES was designed to determine the occurrence and development of eye disorders, including refractive errors, strabismus, amblyopia, and allergic disease, and to identify their environmental and genetic determinants^11–13^. According to previous studies, the prevalence of strabismus ranged from 1.93^14^ to 5.65%^15^ across various regions in China. Assuming a design effect of 2.0, type 1 error of 0.05, and 20% loss to follow-up; a sample size of 4355 children is required to provide over 80% of statistical power to reject the null hypothesis. A total sample of 5000 children were invited.

The sample selection was completed based on a stratified and clustered randomized sampling frame. We stratified all Education Bureau registered primary schools (n = 571) into the 7 cluster regions used by the Hospital Authority Services in Hong Kong. This division into seven clusters is determined by Hong Kong Government according to even distribution of population density in each cluster. Therefore, 714 children should be recruited from each cluster region. The schools in each cluster region were randomly assigned an invitation priority according to the ranking numbers generated by computer. Details of sample size considerations and protocols are described in the previous report^8^. Children attending the territory-wide Hong Kong Children Eye Study from all regions over Hong Kong were invited to this study. All the participating children were ethnic Chinese. Children aged 6 to 8 years old in the sampling frame were included in the study. The exclusion criteria was that children older/ younger than the aforementioned age group were not in the sampling frame. The study adhered to the Declaration of Helsinki. The study protocol was approved by the Ethics Committee Board of the Chinese University of Hong Kong. All children and their parents signed a written informed consent upon their participation in the study.

### Ocular examinations

Distance visual acuity (VA) was measured using a logarithm of the minimum angle of resolution (LogMAR) chart (Nidek, Gamagori, Japan). In the case of uncorrected VA in either eye being > 0.1, best-corrected visual acuity (BCVA) was subsequently measured in a monocular fashion using a trial frame. Refractive status was measured both before and after cycloplegia using an auto-refractor (Nidek ARK-510A, Gamagori, Japan). Two cycles of 1% cyclopentolate (Cyclogyl, Alcon-Convreur, Rijksweg, Belgium) and 1% tropicamide (Santen, Osaka, Japan) were given ten minutes apart. An additional third cycle of cyclopentolate and tropicamide drops was administered thirty minutes after the last drop if either a pupillary light reflex was still present, or pupil size was less than 6.0 mm. Detailed ocular examinations for the anterior segment and the retina were conducted by an ophthalmologist using a slit-lamp (Haag-Streit, Koeniz, Switzerland) and binocular ophthalmoscope with a 20D lens (Volk, Houston, TX) respectively.

### Definition and assessment of ocular alignment

Ocular alignment was taken before cycloplegia with habitual optical correction, if worn. It was evaluated by an ophthalmologist using both the unilateral cover (cover / uncover) test and an alternate cover and prism test. Both tests were administered at near (30 cm) and distance (6 m) fixations, as well as both with and without optical correction if the study subject used any corrective methods. A prism cover test was performed to detect the degree of eye misalignment. A transient misalignment found during alternate cover testing was not defined as strabismus unless confirmed by a repeat unilateral cover test.

Strabismus cases were classified according to the direction of the tropia as esotropic, exotropic, or vertical. A case was considered as a constant tropia if it was constant at both near and distance fixations. Otherwise it was considered as an intermittent tropia. Spherical equivalent (SE) was calculated as the sum of the spherical power plus half of the cylindrical power using cycloplegic refraction. The worse eye was delineated as the eye with the higher absolute value of the SE refractive error.

### Questionnaires

Parents or child carers were asked to complete background information questionnaires, assisted by a trained staff member either in person or over the telephone^8,10^. The information collected included the family demographics and parental data regarding smoking history, alcohol use, the gestational week at delivery, and family histories of eye diseases. Smoking history data was further classified into two types: “maternal smoking during pregnancy” and “maternal smoking,” Each Habitual smoker was asked by the question:” did you quit smoke or continue smoke during pregnancy?” Maternal smoking during pregnancy is defined by whether the mother has ever smoked during pregnancy. Habitual smokers who quit smoke during pregnancy were not defined as “maternal smoking during pregnancy” Regarding gestational data, a low birth weight was defined as being < 2.5 kg, while gestational age was classified for babies born at < 32, 32–36, 37–42, and > 42 weeks respectively^16^. All this information was collected to aid the study in terms of identifying possible risk factors of the development of strabismus among the children in Hong Kong.

### Statistical analysis

Stata (version 14.0, StataCorp LP, College Station, TX) was used for all statistical analyses. Confidence intervals (CIs) and *P* values (significant at levels < 0.05) for prevalence estimates and regression models were calculated with adjustments for cluster effects associated with the sampling design. A multivariate logistic regression was used to investigate the associations of age, gender, and refractive error with strabismus. Risk factors were first explored using a univariate analysis; those which showed at least a marginally significant association (*P* < 0.1) were then considered as candidates for a subsequent forward stepwise multivariate logistic regression. Odds ratios (ORs) and 95% confidence intervals (95% CIs) were calculated to determine significant independent risk factors, which were hence included in the final model.

## Results

### Study population

A total of 4273 children (2236 boys and 2037 girls) responded to the invitation and completed ophthalmological examinations. The response rate was 85.46%. Participating children were 6–8 years old, with a mean (± SD) age 7.61 ± 0.98 years.

### Prevalence and types of strabismus

A total of 133 children (3.11%) were identified as strabismic, with the prevalence being similar across age and gender (*P* = 0.429 and *P* = 0.417 respectively; Table 1). Among them, 117 exhibited concomitant exotropia (63 intermittent, 54 constant-type); 12 exhibited concomitant esotropia; and 4 exhibited purely vertical strabismus. Exotropes outnumbered esotropes by a ratio of 9.75:1. (Table 2) Incomitant deviations were excluded in the analysis. Three children had history of incomitant strabismus. They received treatment due to severe diplopia (1 case) and compensatory head posture (2 cases) and recovered before they attended in the study.

**Table 1:** Prevalence of strabismus in Hong Kong children by age and gender.

	Without strabismus (N)	With strabismus (N)	Prevalence (%)	95% CI (%)		Total (N)	*P* value
	4140	133	3.11%	2.59%	3.63%	4273	
**Age group (years)**							
6	1270	34	2.61%	1.74%	3.47%	1304	0.429
7	1433	51	3.44%	2.51%	4.36%	1484	
8	1437	48	3.23%	2.33%	4.13%	1485	
**Gender**							
Male	2171	65	2.91%	2.21%	3.60%	2236	0.417
Female	1969	68	3.34%	2.56%	4.12%	2037	

*CI* confidence interval.

**Table 2:** Subtypes of Strabismus in Hong Kong children.

Classification	N	Prevalence (%)	95% CI (%)	
Exotropia	117	2.74%	0.63%	1.20%
Esotropia	12	0.28%	0.36%	0.81%
Rate of exotropia: Esotropia	9.75:1			
**Subtypes**				
Intermittent exotropia	63	1.47%	1.11%	1.84%
Constant exotropia	54	1.26%	0.85%	1.49%
Constant esotropia	12	0.28%	0.12%	0.44%
Pure vertical strabismus	4	0.09%	0.00%	0.19%

*CI* confidence interval.

### Associations of strabismus with different types of refractive errors

Strabismus was associated with myopia (≤ − 1.00D; OR 1.72; *P* = 0.012), hyperopia (≥ + 2.00D; OR 2.56; *P* = 0.001), moderate astigmatism (≥ 2.00D; OR 2.33; *P* = 0.048) and severe anisometropia (≥ 2.00 D; OR 2.47; *P* < 0.0001). (Table 3).

**Table 3:** Associations of strabismus with refractive errors.

Refractive status in the worse eye (D)	With Strabismus (N)	Study Subjects (N)	Prevalence (%)	OR#	95% CI (%)		*P* Value
**SE refractive error**							
≤ − 1.00	37	800	4.63%	1.72	1.13	2.62	0.012
> − 1.00 to < + 2.00	75	3174	2.36%	Reference			
≥ + 2.00	21	299	7.02%	2.56	1.50	4.38	0.001
**Astigmatism**							
< 1.00	118	3995	2.95%	Reference			
≥ 1.00 to < 2.00	8	209	3.83%	0.85	0.40	1.81	0.677
≥ 2.00	7	69	10.14%	2.33	1.01	5.37	0.048
**SE anisometropia**							
< 1.00	84	3242	2.59%	Reference			
≥ 1.00 to < 2.00	27	744	3.63%	1.18	0.75	1.86	0.473
≥ 2.00	22	287	7.67%	2.47	1.49	4.09	< 0.001

*SE* spherical equivalent; *D* diopters; *CI* confidence interval; *OR* odds ratio.

^#^Adjusted for age, gender and refractive risk factors in the table in a multivariate logistic regression model.

### Associations of strabismus with parental factors

Among the children in this study, 3959 (92.6%) had a parent or child carer who completed the associated questionnaire. There were no significant differences between the children who had a completed questionnaire and those who did not, in age, gender, SE refractive errors, and prevalence of strabismus. (Supplementary Table S1).

In the univariate analysis, maternal smoking was associated with strabismus. Children whose mothers were habitual smokers were prone to developing strabismus, with prevalence as high as 6.88% (*P* = 0.006). For those children whose mothers smoked during pregnancy, the risk was almost doubled to 11.86% (*P* = 0.002). Other risk factors identified in this analysis included premature birth (*P* = 0.025), advanced maternal age (*P* = 0.015), and family history of strabismus (*P* = 0.001). (Table 4) In the multivariate analysis, strabismus was associated with maternal smoking during pregnancy (OR 4.21; 95% CI 1.80–9.81; *P* = 0.001), family history of strabismus (OR 6.35; 95% CI 2.78–14.50; *P* < 0.0001) and advanced maternal age (OR 1.65, 95% CI 1.09–2.49; *P* = 0.018), and refractive errors (Table 5).

**Table 4:** Univariate analysis for risk factors associated with strabismus in Hong Kong children.

Risk factors	Study subjects (N)	With strabismus		*P value*
		(N)	%	
**Age group**				
6	1215	32	2.63%	0.484
7	1385	47	3.39%	
8	1359	45	3.31%	
Gender (male)	2073	61	2.94%	0.473
Low birth weight (< 2.5 kg)	289	14	4.84%	**0.083**
History of breastfeeding	2652	76	2.87%	0.171
Second-hand smoking exposure after birth	1317	43	3.26%	0.735
Maternal smoking during pregnancy	59	7	11.86%	*0.002
Second-hand smoking exposure during pregnancy	855	33	3.68%	0.168
Maternal smoking	160	11	6.88%	**0.006**
Paternal smoking	1019	31	3.04%	0.848
Alcohol use during pregnancy	102	3	2.94%	*0.911
Maternal age at childbirth > 35 yrs	724	33	4.56%	**0.015**
**Gestational age (weeks)**				
< 32	205	3	1.46%	**0.025**
32–36	3494	108	3.09%	
37–42	235	10	4.26%	
> 42	25	3	12.00%	
Family history of strabismus	44	8	18.18%	* < 0.001
Family history of amblyopia	56	3	5.36%	*0.255
**Socioeconomic factors**				
Household income (< 25,000HKD/month)	1691	53	3.13%	0.778
Maternal education (Secondary school or lower)	2436	82	3.26%	0.419
Paternal education (Secondary school or lower)	2265	65	2.87%	0.475
Housing type PRH or subdivided flats	1265	37	2.92%	0.715

*Fisher exact test.

**Table 5:** Multivariate regression models for independent risk factors of strabismus in Hong Kong children.

Risk factors	OR	95% CI (%)		*P* value
**SE refractive errors**				
≤ − 1.00	1.61	1.03	2.52	0.037
> − 1.00 to < + 2.00	Reference			
≥ + 2.00	2.49	1.42	4.39	0.002
**Astigmatism**				
< 1.00	Reference			
≥ 1.00 to < 2.00	1.09	0.68	1.77	0.716
≥ 2.00	2.32	1.36	3.94	0.002
**SE anisometropia**				
< 1.00	Reference			
≥ 1.00 to < 2.00	0.89	0.41	1.92	0.768
≥ 2.00	3.21	1.36	7.55	0.008
Maternal smoking during pregnancy	4.21	1.80	9.81	0.001
Family history of strabismus	6.35	2.78	14.50	< 0.001
Maternal age at childbirth > 35 years	1.65	1.09	2.49	0.018

Adjusted for age, gender, refractive status and the associated factors (p < 0.1) from univariable analysis in a multivariate logistic regression model.

Based on multivariate stepwise logistic regression model.

*SE* spherical equivalent; *CI* confidence interval; *OR* odds ratio.

## Discussion

In this population-based study of ethnic Chinese school children in the highly urbanized and densely populated city of Hong Kong, we report a prevalence rate of 3.11% for strabismus, which was similar across gender and ages from 6 to 8 years old (Table 1). It is higher than results reported from other populations Japan (6–12 years old, 0.99%)^17,18^, Singapore (0.5–6 years old, 0.84%)^5^, Mexico (2.3%)^19^, and Sydney, Australia (2.8%)^20^ (Supplementary Table S2). MEPEDS previously found that among Asian children with strabismus, exotropia (prevalence, 2.10%; 95% CI 1.44–2.96) was more common than esotropia (prevalence, 1.38%; 95% CI 0.18–2.10)^1^. The exotropia:esotropia ratio was much lower, at 1.5:1^1^. In contrast, most of the strabismic children in the current study had exotropia, 117, while 12 children had esotropia. The prevalence of exotropia was 2.64% (113/4273) and esotropia 0.28% (12/4273), with an exotropia:esotropia ratio of 9.4:1. In STARS conducted in Singapore, where the majority population of Chinese descent is similar to Hong Kong, has shown significantly similar ratio of 7:1^5^. This evidence demonstrates a higher prevalence of exotropia in Chinese population among Asians. Our high prevalence of strabismus could be owing to the high prevalence of myopia in this locality. According to a meta-analysis study conducted in 2016 involving 23,541 subjects, it was found that myopia was an independent risk factor for exotropia with odd ratio 5.23. The study had postulated that the fusional control at distant sight among myopes was weakened; whereas for near vision, owing to the large accommodation lag, less accommodative effort was needed. As the result, less accommodative convergence was stimulated. It was believed that the prolonged suboptimal convergence would cause breakdown of the fusional control and subsequently predispose to the development of exotropia^21^.

Most of the strabismic children in the current study had exotropia, 117 out of 133 (84.96%). The next common was esotropia, 12 out of 133 (9.02%). In this study prevalence of exotropia was 2.64% (113/4273) and esotropia 0.28% (12/4273), with an exotropia:esotropia ratio of 9.4:1. MEPEDS previously found that among Asian children with strabismus, exotropia (prevalence, 2.10%; 95% CI 1.44–2.96) was more common than esotropia (prevalence, 1.38%; 95% CI 0.18–2.10)^1^. The exotropia:esotropia ratio was much lower, at 1.5:1. In STARS, it was concluded that exotropia represented 83.3% of strabismus cases among Singaporean^5^, similar to the 84.96% in Hong Kong Chinese children in this study.

We discovered a significant risk for children with a family history of strabismus, as represented by an OR of 6.35 (95% CI 2.78–14.50, *P* < 0.001). This is consistent with another study conducted in Korea^22^, which quantified the association of family history with strabismus as an OR of 4.91 (95% CI 1.71–14.08; *P* = 0.003). In addition, both MEPEDS^1^ and BPEDS^3^ have also reported a two-fold increase in the risk of developing exotropia among subjects with a family history of strabismus.

Our univariate analysis discovered that maternal smoking, low birth weight, premature birth with a gestational age lower than 32 weeks, and advanced maternal age over 35 years old were all associated with childhood strabismus. In particular, low birth weight, and early gestational age are partly attributed to maternal smoking history, since maternal smoking can give rise to unfavourable pregnancy outcomes^23,6^ including low birth weight^23^ and premature birth^6^. Furthermore, excluding other factors in the stepwise multivariate logistic regression model, maternal smoking during pregnancy was independently highly associated with strabismus with an OR of 4.21. At present, the exact causative mechanism of how prenatal exposure to tobacco would lead to strabismus or poor pregnancy outcomes has not been established. Nevertheless, since the developing foetus is directly exposed to maternal placenta, it is believed that tobacco may induce neurotoxic effects on the foetal nervous system^16^. Greater emphasis should therefore be given to examining how maternal smoking can cause detrimental effects on a child’s vision during pregnancy, including strabismus. Moreover, maternal smoking is a modifiable risk factor, education should be promoted to raise public awareness. On the other hand, future studies should investigate the exact dosage relationship between tobacco smoking and the development of strabismus. Regarding other non-ocular risk factors, advanced maternal age (> 35 years old) carried a *P*-value of 0.018 upon multivariate regression analysis. Although children whose mothers gave birth at an advanced age are generally prone to higher risks of adverse effects, the exact causative mechanism of how advanced age is associated with strabismus is yet to be found.

The associations we found for refractive errors of myopia, hyperopia, astigmatism, and anisometropia with strabismus are generally consistent with other publications^21,24^. A meta-analysis involving 23,541 children showed that myopia resulted in an increased risk of developing concomitant strabismus, and that there was a strong association between hyperopia and concomitant strabismus^8^. In this study, multivariable analysis showed that myopia (*P* = 0.037) and hyperopia (*P* = 0.002) were both associated with strabismus. In addition, astigmatism (≥ 2.00D, *p* = 0.002) and anisometropia (≥ 2.00D, *P* = 0.008) were independently associated with strabismus.

The strengths of our study include its population-based design, relatively large sample size, and standardized methods for sampling and examination. Our study is a territory-wide study and study subjects were from all 18 districts of HK. In addition, all participants were invited to our clinic to receive comprehensive ophthalmological examinations. We maintained proper examination techniques and examination room settings throughout the entire study to achieve high sensitivity and specificity for diagnosing strabismus. These facilities enabled detection of mild cases of strabismus, such as microstrabismus, heterotropia, and heterophoria. In particular, refractive status was measured for all participants both before and after cycloplegia to obtain an accurate assessment of the association between amblyopia and refractive errors.

Findings of our study need to be interpreted with the following caveats. First, this is a cross-sectional study and a causal relationship cannot be concluded. Second, among the 4273 participants who underwent comprehensive ophthalmological examinations, only 3959 had a parent or child carer who accepted the background information questionnaire, reducing the total number of subjects that participated in the risk factor analysis. Third, information on children and families were collected based on the parent-reported questionnaire, which carried a risk of recall bias. Fourth, children who may have been received ophthalmic care from other practicing ophthalmologists, along with those whose strabismus had been completely treated, might have been absent from the study. This may have lowered the prevalence rate. Fifth, we did not include ocular co-morbitidies (except refractive error) or past medical history since the prevalence is relatively low among the healthy subjects of our population based sample group. Instead, we measured other health parameters, including BMI, blood pressure to maximize the yield of comparison. Last but not least, the risk factors of esotropia and exotropia could not be analyzed separately due to insufficient power related to the limited number of cases for each type.

In conclusion, the prevalence of strabismus among ethnic Chinese children age 6–8 years old was found to be 3.11%. Refractive errors are a strong risk factor to strabismus. Among the strabismus cases, exotropia is the predominant type and outnumbers esotropia by a ratio of 9.75:1. Furthermore, family history of strabismus and maternal smoking during pregnancy were significant independent risk factors for strabismus. The adverse effects of maternal smoking on child vision should be emphasized with proper education to parents and public for promotion of child health. In the meantime, efforts should be made through public health service to detect childhood strabismus among the high-risk children whose parents have strabismus or a history of smoking. Early correction of refractive errors and avoidance of maternal smoking during pregnancy are potentially helpful in preventing strabismus.

## Supplementary Information

Supplementary Information.
