# Supplementary material for: Prevalence of strabismus and its risk factors among school aged children: The Hong Kong Children Eye Study
Source: Sci Rep. 2021 Jul 5;11:13820. doi: 10.1038/s41598-021-93131-w (PMC8257606; doi:10.1038/s41598-021-93131-w)
Supplement: Supplementary file 1 — Supplementary Information. [file 41598_2021_93131_MOESM1_ESM.docx]

**Prevalence of strabismus and its risk factors among school-aged children: The Hong Kong Children Eye Study**

Xiu Juan Zhang^1*^, Yi Han Lau^1*^, Yu Meng Wang^1^, Ka Wai Kam^1,2^, Patrick Ip^3^, Wilson W. Yip^1,2^, Simon T. Ko^4,6^, Alvin L. Young^1,2^, Clement C. Tham^1,2,5^, Chi Pui Pang^1^, Li Jia Chen^1,2^, Jason C. Yam^1,2,5,6^

1. Department of Ophthalmology and Visual Sciences, The Chinese University of Hong Kong, Hong Kong SAR, China
2. Department of Ophthalmology and Visual Sciences, Prince of Wales Hospital, Hong Kong SAR, China
3. Department of Paediatrics and Adolescent Medicine, LKS Faculty of Medicine, The University of Hong Kong, Hong Kong SAR, China
4. Department of Ophthalmology, Tung Wah Eastern Hospital, Hong Kong SAR, China
5. Hong Kong Eye Hospital, Kowloon, Hong Kong SAR, China
6. Department of Ophthalmology, Hong Kong Children’s Hospital, Hong Kong SAR, China

**Supplementary Table 1 The Age, Gender, Refraction and Strabismus Distributions between Children who Accepted or Declined the Questionnaires**

|  | **Accepted** | | **Declined** | |  | ***P* value** |
| --- | --- | --- | --- | --- | --- | --- |
|  | **N** | **%** | **N** | **%** |  |  |
| **Age group** |  | | | | | |
| 6 years | 1215 | 28.34% | 89 | 30.69% | 4.341 | 0.114 |
| 7 years | 1385 | 31.53% | 99 | 34.98% |  | |
| 8 years | 1359 | 40.13% | 126 | 34.33% |  | |
| **Gender** |  | | | | | |
| male | 2073 | 52.36% | 163 | 51.91% | 0.024 | 0.878 |
| female | 1886 | 47.64% | 151 | 48.09% |  | |
| **SE Refracitive error** |  | | | | | |
| >−1.00 to <+2.00 | 2944 | 74.36% | 230 | 73.25% | 0.238 | 0.888 |
| ≤−1.00 | 738 | 18.64% | 62 | 19.75% |  | |
| ≥+2.00 | 277 | 7.00% | 22 | 7.01% |  | |
| **Strabismus** |  | | | | | |
| Without | 3835 | 96.87% | 305 | 97.13% | 0.068 | 0.794 |
| With | 124 | 3.13% | 9 | 2.87% |  | |
| **Total** | 3959 | 100.00% | 314 | 100.00% |  | |

SE: spherical equivalent; D: diopters

**Supplementary Table 2 The Prevalence of Strabismus in Reported Studies**

| **Cohort** | **Age (yrs)** | **Prevalence** | **Reference** |
| --- | --- | --- | --- |
| Singapore | 0.5 - 6 | 0.84% | 5 |
| Southwestern China | 7 - 14 | 1.93% | 10 |
| Japan | 6 - 12 | 0.99% | 13,14 |
| Mexico | NA | 2.30% | 15 |
| Sydney, Australia | NA | 2.80% | 16 |
| Rural China | 6 - 14 | 3.53% | 17 |
| Anyang, China | 7 | 5.00% | 18 |
